# Editorial: Mechanistic studies of genome integrity, environmental health, and cancer etiology

**DOI:** 10.3389/fcell.2022.1026326

**Published:** 2022-09-28

**Authors:** Shan Yan, Jianjun Zhao, Michael Kemp, Robert W. Sobol

**Affiliations:** ^1^ Department of Biological Sciences, University of North Carolina at Charlotte, Charlotte, NC, United States; ^2^ Department of Cancer Biology, Lerner Research Institute, Cleveland Clinic, Cleveland, OH, United States; ^3^ Department of Pharmacology and Toxicology, Wright State University, Dayton, OH, United States; ^4^ Mitchell Cancer Institute and Department of Pharmacology, University of South Alabama, Mobile, AL, United States

**Keywords:** genome integrity, environmental health, cancer etiology, DNA repair, DNA damage response

Genomic DNA in all cell types is exposed to insults from endogenous sources, such as oxidative stress, as well as exogenous sources, including environmental genotoxins and anti-cancer therapeutics. Deficiencies in genome integrity maintenance pathways have been implicated in the etiology of cancer and other disease states. To mitigate the debilitating genomic lesions, cells have evolved many different pathways to sense, repair, and signal in response to such challenges. Research studies focused on understanding genome integrity mechanisms have utilized a variety of model organisms and cutting-edge technologies at the molecular, cellular, organismal, and ecological levels. Mechanistic studies that help define the process of genome integrity maintenance, the impact of such mechanisms on environmental health, and their role in cancer etiology are highly significant and have led to new ways of diagnosing and treating cancers and other human diseases. Because of the many advances in this area of research over the past few years, this Research Topic intends to provide the latest insights on the field of genome integrity and to discuss the trends of current and future studies aimed at improving our understanding of disease pathogenesis and treatment. Here, we provide an editorial to summarize these seven research articles and two review articles.

Oxidative stress is a cellular process that is aggravated with aging, by consumption of certain diets, or under chemotherapeutic treatment, and results in damage to the DNA of the cells (Pizzino et al., 2017). Oxidative DNA damage is mainly repaired through the base excision repair (BER) pathway (Maynard et al., 2009). Here, Burchat et al. identified a novel function of human 8-oxoguanine DNA glycosylase-1 (OGG1) beyond its conventional DNA repair function as an initiator of BER-mediated DNA repair related to an increase in tissue mitochondrial content (Figure 1). Further, they reported for the first time that OGG1-mediated obesity resistance in both the Agouti obese (Ay/a) mouse model and the diet-induced obesity (DIO) model requires maternal transmission of the hOGG1 transgene. This novel finding of a critical role for OGG1 in modulating energy balance will open a new research field to connect the conventional DNA repair machinery with mitochondrial function in tissues.

**FIGURE 1:**
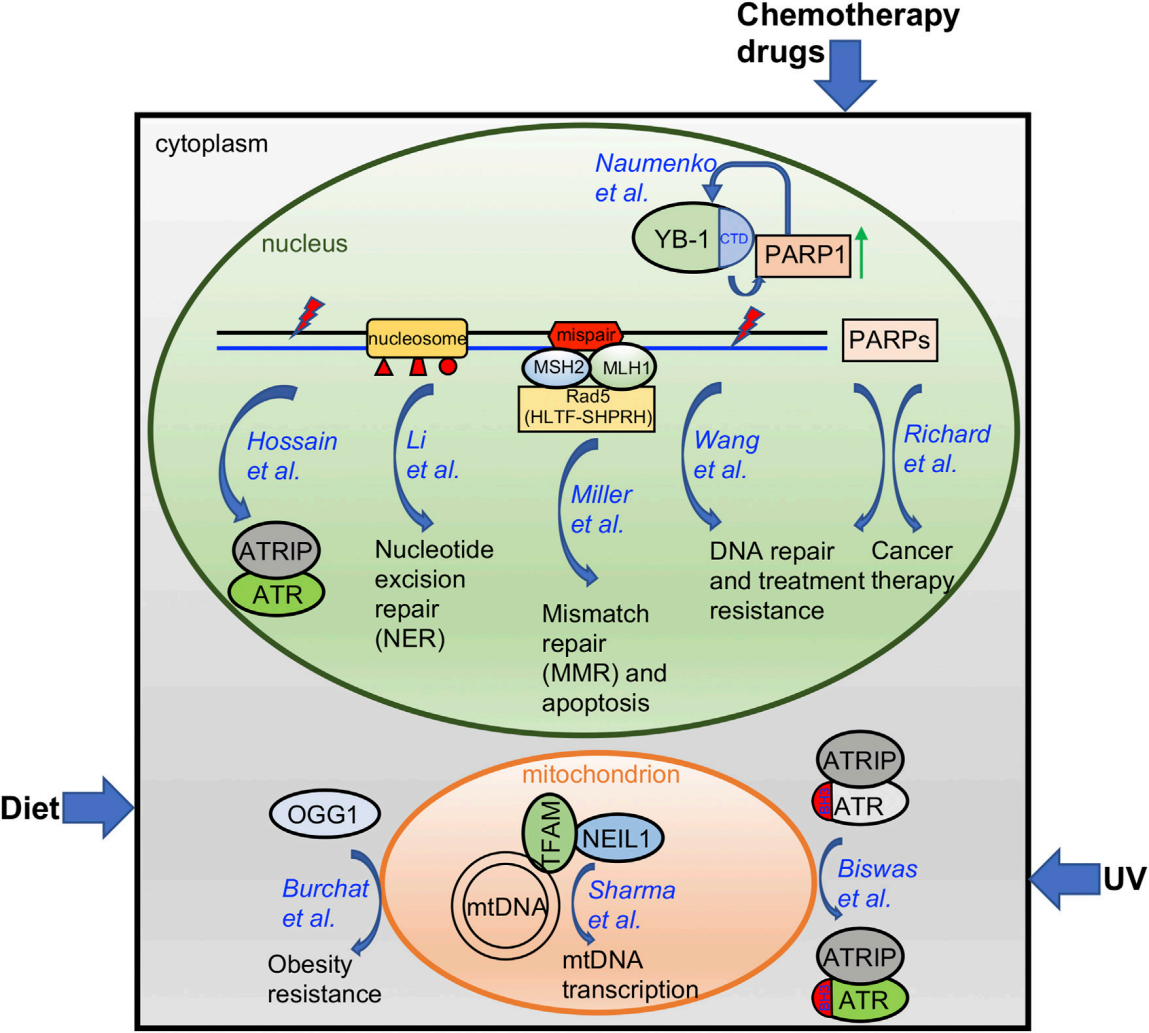
A diagram summarizing the findings from papers included in this Research Topic. Please see text for more details.

Whereas the BER protein Apurinic/Apyrimidinic endonuclease 2 (APE2) has been implicated in the Ataxia-telangectasia and Rad3 related (ATR)-Checkpoint kinase 1 (Chk1) DNA damage response (DDR) pathway in the *Xenopus* system (Willis et al., 2013; Wallace et al., 2017; Lin et al., 2018; Lin et al., 2021), Hossain et al. here provide evidence in pancreatic cancer cells that APE2 is a general regulator of the ATR-Chk1 DDR in response to different stress conditions including oxidative stress, DNA replication stress, and DNA double-strand breaks (DSBs) (Figure 1). A small molecule compound named Celastrol was reported as the first-known APE2 inhibitor that specifically impairs APE2 exonuclease activity by inhibiting its binding to single-stranded DNA (ssDNA). Sensitizing pancreatic cancer cell viability to chemotherapy drugs via APE2-knockdown or exposure to the APE2 inhibitor Celastrol supports the idea that targeting APE2 can provide novel insight into new cancer treatments.

In addition to its well-known checkpoint function in the nucleus, cytoplasmic ATR is converted from *trans*-into a *cis*-isomeric conformation at the Ser428-Pro429 motif within the BH3 domain in a Pin1/DAPK1-regulated manner to suppress apoptosis in mitochondria following ultraviolet (UV) damage (Hilton et al., 2015). Biswas et al. provides the structural basis of the mitochondrial isoform of ATR using a mass spectrometry-based foot printing approach (Figure 1). Two biotin-modified residues K459 and K469 within the BH3 domain of *cis*-ATR are not accessible in *trans-*ATR, suggesting a conformation change around the BH3 domain between *cis*- and *trans*-ATR. Furthermore, cis-ATR with the accessible BH3 domain, but not trans-ATR, is able to associate with tBid. These findings suggest that the isomerization-induced structural changes of mitochondrial specific cis-ATR are essential for its role in cell survival and the DDR pathway.

In recent years, PARP inhibitors (PARPi) targeting PARP1 and PARP2 have been developed as a novel targeted cancer therapeutic due to their roles in DNA damage repair (Javle and Curtin, 2011; Lord and Ashworth, 2017). But many other members of the PARP protein family with a catalytic domain similar to PARP1 and PARP2 are understudied regarding their function on DNA damage repair and tumor initiation (Jubin et al., 2016). Richard et al. comprehensively reviews the current knowledge of the potential functions of PARP isoforms 4 and 6-16 and discusses the roles these proteins may play in DNA damage repair and as targets for cancer therapeutics (Figure 1). This review also points out the future research directions and further research needs to be conducted.

Following the initial treatment of radiation and chemotherapy, cancer recurrence and acquired resistance are major problems in the clinic. Using pairs of same patient-derived primary and recurrent oral cancer cell lines, Wang et al. identified PARP1 upregulation in the recurrent but not primary oral tumor cells and such PARP1 upregulation was augmented by the chemotherapy drugs cisplatin and 5-fluorouracil (Figure 1). Ectopic overexpression of PARP1 rendered the primary cancer cells resistant to chemotherapy drugs and PARP1 inhibitors sensitized recurrent cancer cells to chemotherapy drugs *in vitro* and *in vivo*. Thus, PARP1 upregulation in recurrent oral cancers suggests that targeting PARP1 can be expanded to recurrent oral cancer treatment.

Y-box binding protein 1 (YBX1, YB-1) is a cold shock domain protein that binds both DNA and RNA and is implicated in numerous cellular processes including transcription, translation, mRNA packaging, pre-mRNA splicing and DNA repair. The involvement of YB-1 in these myriad mechanisms are mediated via numerous protein-protein interactions. Similarly, its role in DNA repair involves interactions with MSH2, DNA polymerase delta, Ku80 and WRN (Gaudreault et al., 2004), RAD21 (Panigrahi et al., 2012), BARD1 and BRCA1 (Woods et al., 2012). Recently, YB-1 was identified among proteins proximal to trapped PARP1 (Krastev et al., 2022). This new report by Naumenko et al. provides more detail on the interaction between YB-1 and PARP1 and the role of YB-1 in regulating poly-ADP-ribose (PAR) synthesis via the interaction between the disordered YB-1 C-terminal domain (CTD) and PAR (Figure 1). Overall, they suggest that YB-1 CTD-like domains may be considered PAR “readers” similar to other known PAR-binding modules.

Nucleotide excision repair (NER) plays an essential role in the removal of bulky DNA lesions induced by UV radiation and other genotoxins. Though biochemical studies have defined the NER mechanism on naked DNA *in vitro*, the packaging of DNA into histones and higher order chromatin structures in the cell *in vivo* likely impacts the ability of the NER machinery to do its job. In this perspective, Li et al. provides a comprehensive summary of how different post-translational modifications to histones by specific classes of enzymes impact NER function *in vivo* (Figure 1).

Proteins of the mismatch repair (MMR) pathway function to correct replication errors including base-base mispairs and small insertion/deletion mispairs (Jiricny, 2006; Li, 2008; Fishel, 2015). In addition, the MMR pathway recognizes DNA damage induced mispairs to trigger apoptosis, such as that induced by the O^6^-methylguanine:thymidine mispairs that arises after exposure to SN1 alkylators (Fu et al., 2012; Soll et al., 2017; Fujii et al., 2022). While the core proteins for MMR have been well defined (Modrich, 2016), additional proteins that complex with MMR proteins and that may regulate the MMR pathway are anticipated. Here, Miller et al. identified Rad5 (*Saccharomyces cerevisiae*) as an Mlh1 and Msh2 interacting protein (Figure 1). Further, they show that the human counterparts of Rad5 (HLTF and SHPRH) interact with MSH2 and MLH1, respectively. This novel finding will form the basis for future studies to uncover the detailed functional role of these and other MMR interacting proteins.

In response to constant endogenous and/or exogenous sources of DNA damaging agents, it is essential for cells to maintain the stability of the 16.5 kb mitochondrial DNA (mtDNA) in humans, in addition to the nuclear genome (Copeland and Longley, 2014). Sharma et al. here reported and characterized the interaction between the BER protein Nei-like DNA Glycosylase 1 (NEIL1) and mitochondrial transcription factor A (TFAM) which requires the presence of DNA/RNA (Figure 1). Interestingly, NEIL1 is necessary for efficient transcription by TFAM upon alkylating agent induced DNA damage. The regulation of the NEIL1-TFAM interaction by salt concentrations, protein availability, nucleic acids, and the presence of DNA damage suggests a transient, dynamic, and functional association to maintain mtDNA stability.

Overall, the research and review articles in this Research Topic have identified and/or characterized several distinct mechanisms of how cells respond to environmental factors, such as diet, UV, and chemotherapy drugs, and how nuclear genome and epigenome integrity and mitochondria stability are maintained (Figure 1). Future studies in the field to consider include 1) how different environmental factors including but not limited to air pollutants, viruses, and pesticides affect genomic and/or epigenomic integrity; 2) how distinct DNA repair and DDR pathways sense and signal environmental and intrinsic insults; 3) how cutting-edge omics technologies are applied to better understand genome integrity and public health; and 4) how our novel knowledge in genome/epigenome integrity provides better strategies for cancer therapeutics.

## References

[B1] Copeland W. C. Longley M. J. (2014). Mitochondrial genome maintenance in health and disease. DNA Repair (Amst) 19, 190–198. 10.1016/j.dnarep.2014.03.010 24780559 PMC4075028

[B2] Fishel R. (2015). Mismatch repair. J. Biol. Chem. 290, 26395–26403. 10.1074/jbc.R115.660142 26354434 PMC4646297

[B3] Fu D. Calvo J. A. Samson L. D. (2012). Balancing repair and tolerance of DNA damage caused by alkylating agents. Nat. Rev. Cancer 12, 104–120. 10.1038/nrc3185 22237395 PMC3586545

[B4] Fujii S. Sobol R. W. Fuchs R. P. (2022). Double-strand breaks: When DNA repair events accidentally meet. DNA Repair (Amst) 112, 103303. 10.1016/j.dnarep.2022.103303 35219626 PMC8898275

[B5] Gaudreault I. Guay D. Lebel M. (2004). YB-1 promotes strand separation *in vitro* of duplex DNA containing either mispaired bases or cisplatin modifications, exhibits endonucleolytic activities and binds several DNA repair proteins. Nucleic Acids Res. 32, 316–327. 10.1093/nar/gkh170 14718551 PMC373280

[B6] Hilton B. A. Li Z. Musich P. R. Wang H. Cartwright B. M. Serrano M. (2015). ATR plays a direct antiapoptotic role at mitochondria, which is regulated by prolyl isomerase Pin1. Mol. Cell 60, 35–46. 10.1016/j.molcel.2015.08.008 26387736 PMC4592481

[B7] Javle M. Curtin N. J. (2011). The role of PARP in DNA repair and its therapeutic exploitation. Br. J. Cancer 105, 1114–1122. 10.1038/bjc.2011.382 21989215 PMC3208503

[B8] Jiricny J. (2006). The multifaceted mismatch-repair system. Nat. Rev. Mol. Cell Biol. 7, 335–346. 10.1038/nrm1907 16612326

[B9] Jubin T. Kadam A. Jariwala M. Bhatt S. Sutariya S. Gani A. R. (2016). The PARP family: Insights into functional aspects of poly (ADP-ribose) polymerase-1 in cell growth and survival. Cell Prolif. 49, 421–437. 10.1111/cpr.12268 27329285 PMC6496725

[B10] Krastev D. B. Li S. Sun Y. Wicks A. J. Hoslett G. Weekes D. (2022). The ubiquitin-dependent ATPase p97 removes cytotoxic trapped PARP1 from chromatin. Nat. Cell Biol. 24, 62–73. 10.1038/s41556-021-00807-6 35013556 PMC8760077

[B11] Li G. M. (2008). Mechanisms and functions of DNA mismatch repair. Cell Res. 18, 85–98. 10.1038/cr.2007.115 18157157

[B12] Lin Y. Bai L. Cupello S. Hossain M. A. Deem B. McLeod M. (2018). APE2 promotes DNA damage response pathway from a single-strand break. Nucleic Acids Res. 46, 2479–2494. 10.1093/nar/gky020 29361157 PMC5861430

[B13] Lin Y. McMahon A. Driscoll G. Bullock S. Zhao J. Yan S. (2021). Function and molecular mechanisms of APE2 in genome and epigenome integrity. Mutat. Res. Rev. Mutat. Res. 787, 108347. 10.1016/j.mrrev.2020.108347 34083046 PMC8287789

[B14] Lord C. J. Ashworth A. (2017). PARP inhibitors: Synthetic lethality in the clinic. Science 355, 1152–1158. 10.1126/science.aam7344 28302823 PMC6175050

[B15] Maynard S. Schurman S. H. Harboe C. de Souza-Pinto N. C. Bohr V. A. (2009). Base excision repair of oxidative DNA damage and association with cancer and aging. Carcinogenesis 30, 2–10. 10.1093/carcin/bgn250 18978338 PMC2639036

[B16] Modrich P. (2016). Mechanisms in *E. coli* and human mismatch repair (nobel lecture). Angew. Chem. Int. Ed. Engl. 55, 8490–8501. 10.1002/anie.201601412 27198632 PMC5193110

[B17] Panigrahi A. K. Zhang N. Otta S. K. Pati D. (2012). A cohesin-RAD21 interactome. Biochem. J. 442, 661–670. 10.1042/BJ20111745 22145905

[B18] Pizzino G. Irrera N. Cucinotta M. Pallio G. Mannino F. Arcoraci V. (2017). Oxidative stress: Harms and benefits for human health. Oxid. Med. Cell. Longev. 2017, 8416763. 10.1155/2017/8416763 28819546 PMC5551541

[B19] Soll J. M. Sobol R. W. Mosammaparast N. (2017). Regulation of DNA alkylation damage repair: Lessons and therapeutic opportunities. Trends biochem. Sci. 42, 206–218. 10.1016/j.tibs.2016.10.001 27816326 PMC5336464

[B20] Wallace B. D. Berman Z. Mueller G. A. Lin Y. Chang T. Andres S. N. (2017). APE2 Zf-GRF facilitates 3'-5' resection of DNA damage following oxidative stress. Proc. Natl. Acad. Sci. U. S. A. 114, 304–309. 10.1073/pnas.1610011114 28028224 PMC5240719

[B21] Willis J. Patel Y. Lentz B. L. Yan S. (2013). APE2 is required for ATR-Chk1 checkpoint activation in response to oxidative stress. Proc. Natl. Acad. Sci. U. S. A. 110, 10592–10597. 10.1073/pnas.1301445110 23754435 PMC3696815

[B22] Woods N. T. Mesquita R. D. Sweet M. Carvalho M. A. Li X. Liu Y. (2012). Charting the landscape of tandem BRCT domain-mediated protein interactions. Sci. Signal. 5, rs6. 10.1126/scisignal.2002255 22990118 PMC4064718

